# Biomarkers of Genotoxicity in Medical Workers Exposed to Low-Dose Ionizing Radiation: Systematic Review and Meta-Analyses

**DOI:** 10.3390/ijms22147504

**Published:** 2021-07-13

**Authors:** Clémence Baudin, Marie-Odile Bernier, Dmitry Klokov, Maria Grazia Andreassi

**Affiliations:** 1 Ionizing Radiation Epidemiology Laboratory, Institute for Radiological Protection and Nuclear Safety, 92262 Fontenay-aux-Roses, France; marie-odile.bernier@irsn.fr; 2 Experimental Radiotoxicology and Radiobiology Laboratory, Institute for Radiological Protection and Nuclear Safety, 92262 Fontenay-aux-Roses, France; dmitry.klokov@irsn.fr; 3 Department of Biochemistry, Microbiology and Immunology, University of Ottawa, Ottawa, ON K1N 6N5, Canada; 4 CNR Institute of Clinical Physiology, 56124 Pisa, Italy; andreas@ifc.cnr.it

**Keywords:** systematic review, meta-analysis, medical workers, ionizing radiation, cytogenetic biomarkers, DNA integrity

## Abstract

Medical staff represent the largest group of workers occupationally exposed to ionizing radiation (IR). Chronic exposure to low-dose IR may result in DNA damage and genotoxicity associated with increased risk of cancer. This review aims to identify the genotoxicity biomarkers that are the most elevated in IR-exposed vs. unexposed health workers. A systematic review of the literature was performed to retrieve relevant studies with various biomarkers of genotoxicity. Subsequent meta-analyses produced a pooled effect size for several endpoints. The search procedure yielded 65 studies. Chromosome aberrations (CA) and micronuclei (MN) frequencies were significantly different between IR-exposed and unexposed workers (θ_pooled_ = 3.19, 95% CI 1.46–4.93; and θ_pooled_ = 1.41, 95% CI 0.97–1.86, for total aberrant cells and MN frequencies, respectively), which was not the case for ring chromosomes and nucleoplasmic bridges. Although less frequently used, stable translocations, sister chromatid exchanges (SCE) and comet assay endpoints were also statistically different between IR-exposed and unexposed workers. This review confirms the relevance of CA and MN as genotoxicity biomarkers that are consistently elevated in IR-exposed vs. unexposed workers. Other endpoints are strong candidates but require further studies to validate their usefulness. The integration of the identified biomarkers in future prospective epidemiological studies is encouraged.

## 1. Introduction

Exposure of humans to ionizing radiation (IR) during medical procedures is the greatest contributor to annual radiation exposure dose from all artificial IR sources. Diagnostic and therapeutic use of IR has substantially increased in the last decades, exemplified by a range of new imaging techniques [1,2] and new targeted irradiation therapeutic modalities [3]. Accordingly, medical workers comprise the largest professional human group that are exposed to occupational IR at low doses and low dose-rates (7.35 million worldwide, representing 75% of workers exposed to artificial sources of radiation) [4]. Exposure to moderate-to-high doses of IR induces genotoxic effects that can lead to carcinogenesis [5]. However, whether such detrimental effects can be produced after exposure to low-dose IR is still debated [6]. To this end, the value of biomarkers has been recognized as a complementary tool to conventional epidemiology that can facilitate understanding the health effects of low-dose IR exposures [7]. They may fill important gaps in the understanding of the biological mechanisms that link IR exposure and disease. IR induces many types of deoxyribonucleic acid (DNA) lesions, of which DNA double-strand breaks (DSB) are recognized as the ones that have the greatest role in radiation-induced genomic instability and subsequently carcinogenesis [8,9]. Unrepaired or mis-repaired DSB can lead to the formation of chromosome aberrations (CA), a broad class of DNA mutations that are linked to various health risks [10]. Increased rates of CA in peripheral blood lymphocytes (PBL) have been associated with an increased risk of cancer [11,12,13]. CA can therefore be considered as potential bioindicators of cancer risk.

Numerous types of CA can be broadly categorized into stable (e.g., inversions and translocations) and unstable (e.g., acentric fragments, dicentrics, and rings) aberrations. The former are non-lethal for cells and can persist for years, whereas the latter cause cell death during mitosis, thus are considered short lived genotoxic events that decline with time after the triggering insult [10]. Detection and quantification of unstable CA is simple and employed shortly after IR exposure, e.g., for biodosimetry [14]. Persistence of stable CA allows for the evaluation of the long-term effects of single IR exposures or the cumulative effects of protracted chronic exposures [15]. However, the detection of this type of CA requires more elaborated and costly techniques, such as fluorescence in situ hybridization (FISH). Sister chromatid exchanges (SCE) are reciprocal exchange of segments between two identical sister chromatids, resulting from damaged DNA and involving several mechanisms during the S-phase. SCE are frequently reported as a marker of spontaneous and induced chromosomal instability in cells [16]. Although acute IR exposure is not efficient in producing SCE [17], increased SCE levels were seen in populations chronically exposed to IR [18]. Micronuclei (MN) originate from chromosome fragments or whole chromosomes that, upon nuclear division, lag behind the anaphase and are not properly segregated into daughter cell nuclei. Therefore, they represent chromosomal instability and can also serve as an indicator of chromosomal damage induced by IR [19]. Additionally, MN frequency was shown to be strongly correlated with the length of telomeres (the terminal structures of linear chromosomes), whose role is to protect chromosomes and participate in the integrity of the genetic heritage [20]. Interestingly, recent studies show that IR exposure causes telomere length shortening in Chernobyl accident recovery workers that could potentiate carcinogenesis [21]. Thus, the measurement of telomere lengths in people exposed to IR bears a potential as a biomarker of the risk of cancer and other age-related diseases [22]. Molecular damage to DNA, such as single-strand breaks (SSB) and DSB, can also be measured using various assays such as the comet assay for SSB and the detection of immunofluorescently labeled phosphorylated H2AX (designated as γH2AX) foci.

Although there exist a great number of studies examining genotoxicity biomarkers in IR-exposed medical professionals, results are often inconsistent or conflicting [23,24]. These studies often employ small size human cohorts causing low statistical power to discriminate IR-exposed and unexposed workers and discrepancies in results. This provides motivation for conducting a quantitative meta-analysis of the published results in an attempt to summarize and analyze the available knowledge and to reveal the most sensitive and reliable biomarkers. Therefore, the objective of the present work was to carry out a systematic review and meta-analysis to identify the type(s) of genotoxicity biomarkers that are most elevated in IR-exposed compared to unexposed medical workers. These biomarkers, as most sensitive to IR exposure, would then provide a possibility for future focused prospective epidemiological studies to examine the association between these biomarkers and long-term health outcomes, primarily cancer.

## 2. Methods

These systematic review and meta-analyses have been made according the PRISMA guidelines, as a basis for reporting systematic reviews. Registration has been recorded on the PROSPERO database (ID CRD42020182636).

### 2.1. Online Searches

The literature search was conducted in the PubMed, Google Scholar, Scopus and Web of Science databases, in April 2020. The following query has been used: (((ionizing radiation) AND medical workers) AND (chromosome OR biomarker OR genetic OR DNA)). Relevant publications and international reports such as BEIR VII and UNSCEAR 2006 were also screened for additional references. Duplicates from the different databases were removed. Based on the results of this research, a first selection was conducted by two independent reviewers (MGA and CB) who reviewed all titles and abstracts according to predefined selection criteria (see below), and disagreements were resolved by a third reviewer (MOB). A second selection was conducted based on full-text screening. For all articles eventually included in the review, relevant information was collected including study title, first author, journal, year of publication, study design, population, inclusion and exclusion criteria, sample size, objectives, and endpoints. The systematic literature review was then supplemented by meta-analyses.

### 2.2. Selection Criteria

To be eligible, studies had to be observational (longitudinal/cohort, case-control or cross-sectional), in English, and published between 1 January 2000 and 31 March 2020, The rational for the use of this time range was the marked growth of the nuclear medicine domain and the development of new and the refinement of old genotoxicity biomarkers and their detection. Studies with 15 participants or less in one of the exposed/unexposed groups were excluded. The meta-analyses included only the studies with common and complete quantitative information (measurement data of the evaluated endpoint such as frequencies, length or score, for both IR-exposed and unexposed workers groups) where only studies with exposed/unexposed design were considered.

Selection criteria for relevant studies have been made following the PECO statements (Table 1).

The target population (P) were all medical radiation workers potentially exposed to IR: radiological technologists, radiologists, interventional cardiologists, nurses and laboratory technicians.

The exposure (E) comprised IR from all medical sources (external irradiation or internal contamination). When available, dose estimates were reported either as absorbed doses to organs (mGy) or effective doses (mSv). Otherwise, surrogates were also considered (occupational radiological risk score (ORRS), index of cumulative radiological score, number of years at exposed work, reconstruction of the lifetime cumulative professional exposure, etc.).

Comparisons (C) between exposed and unexposed workers, or between different categories of exposure were studied.

For the outcomes (O), this review focused on biomarkers in peripheral blood lymphocytes: cytogenetic biomarkers such as CA (stable and unstable: chromosome and/or chromatid breaks, inversions, insertions, deletion, gaps, aneuploidy, dicentrics, acentric fragments, rings, and translocations), MN, nucleoplasmic bridges (NPB), SCE, or premature centromere divisions (PCD), and markers of DNA integrity such as mutated gene frequencies, γH2AX foci, comet tail length/moment, telomere length (TL), and DNA strand breaks. As most of the CA studies presented percent or fraction of aberrant cells or number of cells with at least one CA, the total of aberrant cells has also been analyzed making it possible to pool results from studies using different CA types for the purpose of meta-analysis. Studies involving oxidative stress and inflammation biomarkers, apoptosis, cell cycle, and gene expression biomarkers, or genetic susceptibility biomarkers (influence of SNPs on biomarkers of effect or cancer risk) were excluded.

### 2.3. Quality Assessment

To assess the quality of the included studies, the Newcastle–Ottawa Scale was used, which is the tool most commonly used nowadays for observational studies. The assessment is based on eight items categorized into three groups: selection of study groups, comparability of groups and determination of exposure or outcome of interest for case-control or cohort/cross-sectional studies. A study can be assigned a maximum of one star for each of the eight items (up to two stars for the comparability group item). A final score between 0 and 9 is obtained by adding up all the stars. Studies with a score <3 were excluded.

### 2.4. Statistical Analysis

Since most studies presented continuous variables for biomarkers (frequencies, comet tail or telomere length, etc.) in both IR-exposed and unexposed workers, we computed Hedges’s g [25] standardized mean differences for individual studies. Briefly, Hedges’s g is defined as the unbiased difference between two means (m1 and m2) divided by a pooled weighted standard deviation (s* calculated from standard deviations s1 and s2) for two populations to be compared (population sizes *n*1 and *n*2):

$$g=\left(1-\frac{3}{4\left(n1+n2\right)-9}\right)\times \frac{m1-m2}{s*}$$

with $s*\;=\surd \frac{\left(n1-1\right)s1^{2}+\left(n2-1\right)s2^{2}}{n1+n2-2}$.

Then, standardized mean differences were pooled together to generate and plot an overall effect size using the DerSimonian–Laird random-effect method, involving the assumption that the effects estimated in the different studies are not equal. A Z-test was used to assess the null hypothesis whereby the overall effect size would not be significantly different from 0. The between-study heterogeneity was reported using the Cochrane’s homogeneity test (Q) and the I^2^ statistic which allows to quantify the proportion of the total variation due to that heterogeneity [26]. Finally, small-study effects and publication bias were visually and numerically explored using Egger’s test.

Data were analyzed using Stata 16 software (StataCorp. 2019. Stata Statistical Software: Release 16. StataCorp LLC: College Station, TX, USA) using the *meta* command. Statistical significance was defined by *p* < 0.05.

## 3. Results

**Figure 1:**
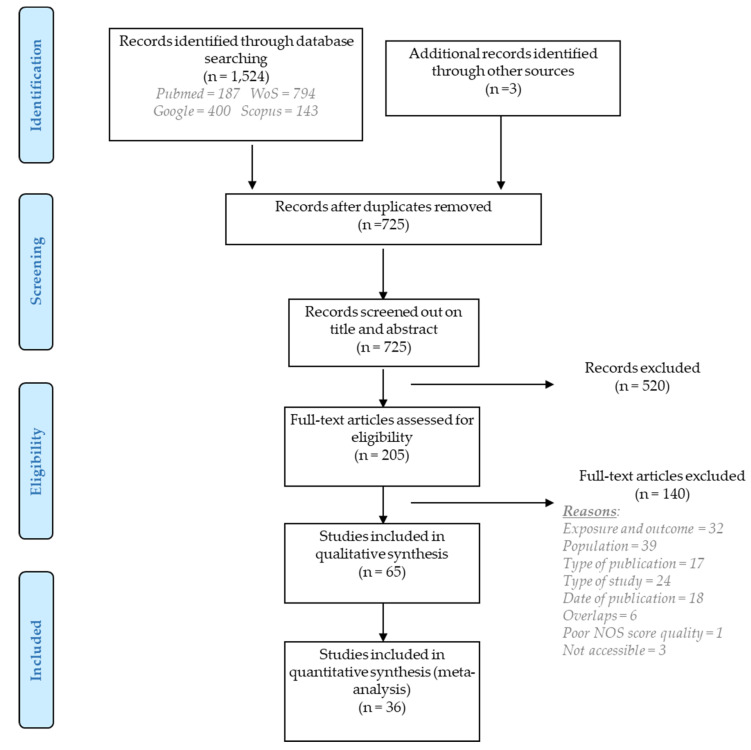
PRISMA flow diagram for the results of literature search, screening, and selection of relevant studies.

The systematic search produced 1524 records. Three additional records were identified from reference screening in relevant papers or reports. After removing duplicates, 725 titles and abstracts were screened using PECO, and eventually 205 articles were selected for a full-text analysis. Exclusion of 134 articles based on full-text screening resulted in 65 studies suitable for inclusion in the qualitative synthesis. Of those 65, 36 articles contained sufficient information and data to be included in a quantitative meta-analysis (Figure 1).

Reasons for exclusion after full-text evaluation included: exposure and outcome did not meet the PECO criteria (*n* = 32), the population exposed to IR did not involve medical workers or was <15 in number (*n* = 39), some publications were books/conference proceedings/systematic reviews (*n* = 17), the type of study was experimental (*n* = 24), the NOS quality assessment was <3 (*n* = 1), the articles were published before 2000 (*n* = 18) or were not accessible (*n* = 3) or overlapped with others (*n* = 6). For the purpose of maintaining structured presentation of results, the various endpoints were grouped into two large categories: cytogenetic and DNA integrity biomarkers.

### 3.1. Cytogenetic Biomarkers

Fifty-three out of the 65 studies included in the systematic review investigated cytogenetic endpoints to examine differences between IR-exposed and non-exposed professionals. CA frequencies was the focus of 30 studies, including at least dicentrics, acentric fragments, and/or rings for 24, 14, and 14 studies respectively. Out of the 65 studies included in the present systematic review, MN frequencies, NPB, SCE and PCD were the focus of 32, 7, 7, and 2 studies respectively.

**Figure 2:**
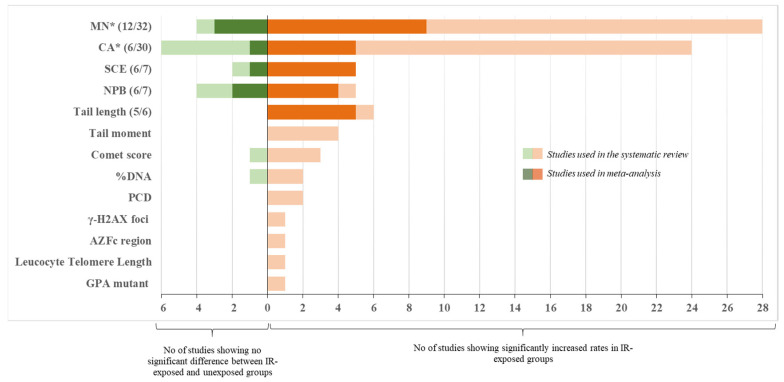
Summary chart of the included studies. Shown are numbers of studies broken by endpoint. Studies that reported significant increase in endpoints in IR-exposed groups are plotted to the right of the X = 0, whereas studies showing no difference are plotted to the left. Studies used in meta-analysis are shown in dark color and their numbers are also shown in the brackets (e.g., 6/7 means 6 out of 7 total studies qualified and were used in meta-analysis). * Correlation of the endpoint with other confounding factors (alcohol consumption, smoking) were reported (see text for detail). AZFc: azoospermia factor region c; CA: chromosome aberration; GPA: glycophorin A; MN: micronucleus; NPB: nucleoplasmic bridge; PCD: premature centromere divisions; SCE: sister chromatid exchange.

Overall, most of the studies reported significantly higher frequencies of CA frequencies in IR-exposed compared to unexposed workers (Table 2, Figure 2).

**Figure 3:**
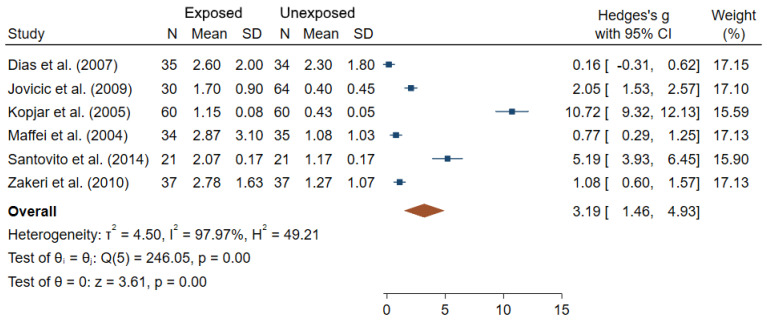
Forest plot of mean differences in fraction of cells with CA between IR exposed and unexposed workers. The blue squares represent the differences in standardized means between IR-exposed and non-exposed workers for each study individually (with their associated blue bar corresponding to the confidence interval of each mean), while the brown diamond below corresponds to the estimated overall standardized mean difference (placed in the center of the diamond, with the bounds of the confidence interval being at the extreme points of the diamond).

Few studies investigated dose-response analyses between low-dose IR exposure and CA frequencies in medical workers, with no clear relationships reported [53,55,90]. In Figure 3, results of the six studies with complete information are summarized in a form of a Forest plot showing the differences in the rates of aberrant cells between IR-exposed and unexposed workers [35,48,52,57,73,88]. An estimated overall standardized mean difference (θpooled = 3.19; 95% CI 1.46–4.93) was significantly different from 0 (Z = 3.61, *p* < 10^−3^). A high heterogeneity between studies was observed (I^2^ = 97.97%; Q = 246.05, *p* < 10^−3^). Small-study effects and publication bias were found using the Egger’s test (*p* < 10^−3).^

**Figure 4:**
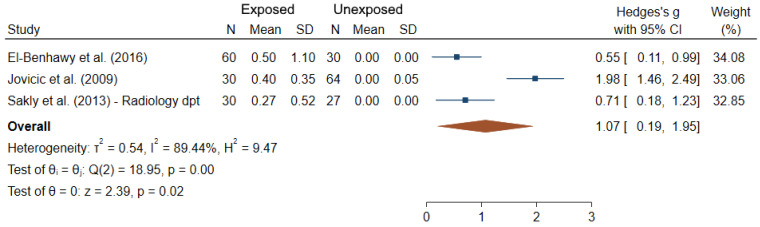
Forest plot of mean differences in dicentrics between IR exposed and unexposed workers.

Among the 24 studies which focused on dicentrics, only three provided sufficient data for meta-analysis (Figure 4). It can be noted that the rates of dicentrics in controls were zero; and the mean difference between IR-exposed and unexposed workers was significantly greater than 0 (θpooled = 1.07; 95% CI 0.19–1.95; Z = 2.39, *p* = 0.02), results being heterogeneous between studies (I^2^ = 89.44%; Q = 18.95, *p* < 10^−3^), but the Egger regression-based test did not show publication bias and small-study effects (*p* = 0.61).

**Figure 5:**
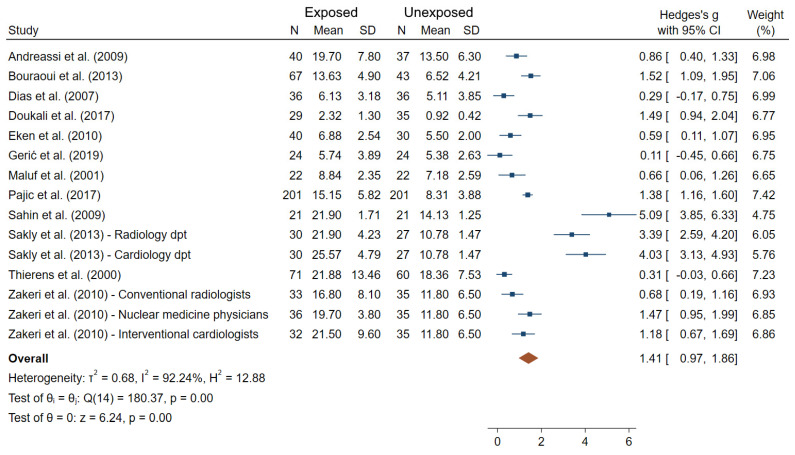
Forest plot of mean differences in MN frequencies between IR exposed and unexposed workers.

With respect to the studies dealing with MN frequencies, which is the most widely used endpoints among studies, 28 out of the 32 did show significantly higher frequencies in exposed compared to unexposed workers using comparisons tests between groups (Figure 2). Additionally, among the 32 MN studies, 23 evaluated the relationship (correlation or dose-response) between IR exposure and MN frequencies using two alternative indicators of exposure (using one or both indicators): cumulative dose from personal dosimeters (14 studies) and/or duration of exposure in years (16 studies). Among those, a significant association/correlation between cumulative dose or duration of exposure and MN frequencies were reported in 5/14 and 9/16 studies, respectively [23,32,38,61,64,65,74,77,82]. Additionally, in professionals working in a nuclear medicine department, both MN and SCE levels were significantly higher during their occupational exposure compared to levels immediately after vacation period [70]. Twelve out of 32 studies satisfied the criteria for inclusion into meta-analysis. Figure 5 shows an overall mean difference of MN frequencies comparisons between IR-exposed and unexposed workers (θpooled = 1.41; 95% CI 0.97–1.86) which was significantly different from 0 (Z = 6.24, *p* < 10^−3^), with high heterogeneity between study-specific effect sizes (I^2^ = 92.24%; Q = 180.37, *p* < 10^−3^). The Egger regression-based test revealed small-study effects and publication bias (*p* < 10^−3^).

**Figure 6:**
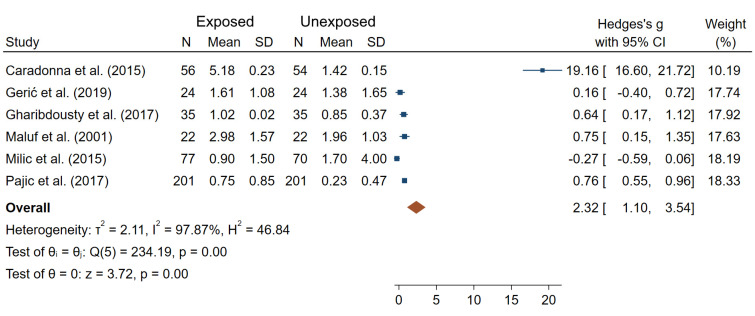
Forest plot of mean differences in NPB frequencies between IR exposed and unexposed workers.

In terms of NPB frequencies, four studies reported a lack of a significant difference between IR-exposed and unexposed workers [24,45,61,69], and three studies showed significantly elevated NPB frequencies in IR-exposed medical workers compared to controls [33,58,65]. Meta-analysis carried out on the data from six studies with complete information (Figure 6) reported a significant overall difference in NPB frequencies between IR exposed and unexposed workers (θpooled = 2.32; 95% CI 1.10–3.54; Z = 3.72, *p* < 10^−3^; I^2^ = 97.87%; Q = 234.19, *p* < 10^−3^). Small-study effects and publication bias (*p* < 10^−3^) were observed.

The mean number of SCE per cell was significantly higher in five out of seven studies. While SCE number was significantly higher in a group of workers with a duration of employment ≥15 years compared to a group with <15 years of employment [38], only two studies conducted linear regression and reported a positive but non-significant β-coefficient for SCE frequency with an increase in IR exposure assessed by duration of employment [73] or exposure dose [39].

**Figure 7:**
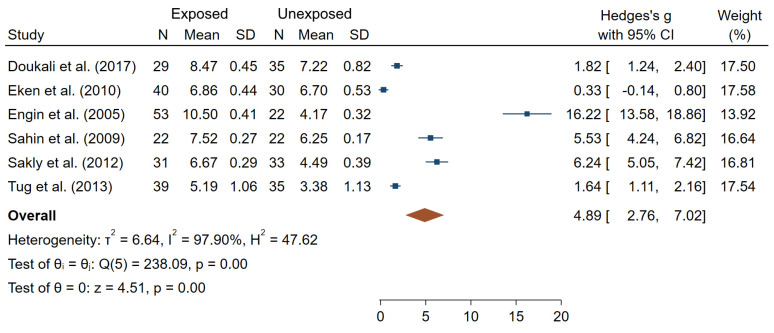
Forest plot of mean differences in SCE frequencies between IR exposed and unexposed workers.

Figure 7 reports overall mean differences in SCE frequencies between IR-exposed and unexposed workers for the six studies included in meta-analysis (θpooled = 4.89; 95% CI 2.76–7.02), which was significantly different from 0 (Z = 4.51, *p* < 10^−3^). High heterogeneity (I^2^ = 97.90%; Q = 238.09, *p* < 10^−3^), small-study effects and publication bias were observed (*p* < 10^−3^).

Lastly, the only two studies that focused on PCD reported significantly higher frequencies in IR-exposed workers compared to unexposed, regardless of a specific PCD readout used [49,64].

### 3.2. DNA Integrity Biomarkers

Only 18 out of the 65 studies evaluated various DNA integrity endpoints as genotoxicity biomarkers. These were comet tail length (TL; six studies), comet tail moment (TM; four studies), comet score (DNA damage extent, CS; four studies), %DNA in the tail (%DNA; three studies), DNA strand breaks (SBs; one study), glycophorin A (GPA) mutant (one study), leucocyte telomere length (LTL; one study), copy number variation in AZFc region (one study), and γ-H2AX foci (1 study), with almost no overlap within a single study.

**Figure 8:**
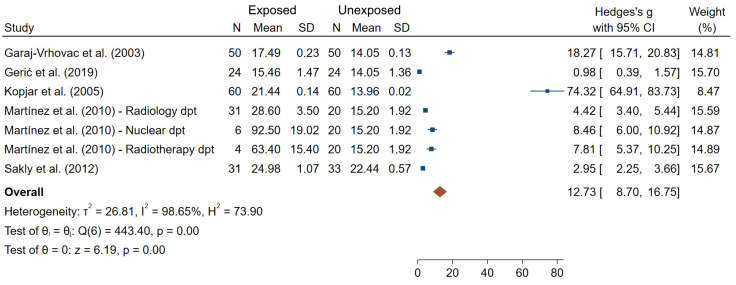
Forest plot of mean differences in tail length between IR exposed and unexposed workers.

TL was significantly greater in IR-exposed compared to unexposed workers in all the six studies (out of the six reporting results for this endpoint). Additionally, TL was found to increase significantly at the end of a work day in IR-exposed individuals, but not in unexposed workers [59]. It can also be noted that differences in TL were found between various hospital departments and working places; however, no common pattern across studies was seen [44,52,59,82]. Figure 8 presents an overall mean difference for TL between IR-exposed and unexposed workers (θpooled = 12.73; 95% CI 8.70–16.75) which was significantly different from 0 (Z = 6.19, *p* < 10^−3^). However, large heterogeneities (I^2^ = 98.65%; Q = 443.40, *p* < 10^−3^), small-study effects and publication bias (*p* < 10^−3^) have been observed.

A higher %DNA in the comet tail was reported in IR-exposed compared to unexposed workers (statistically significant in two studies [37,42], and non-significant in one study [45]).

TM, which is an integral damage parameter derived from TL and %DNA, was found to be significantly higher in IR-exposed workers compared to unexposed workers in all 4 studies reporting TM [37,42,44,82]. Whereas no correlation between TM or %DNA and effective yearly dose measured by individual dosimeters was observed in nuclear medicine workers [37], an increased gradient in TM and in %DNA was found according to a 10-year increase in X-ray exposure duration [42].

Assigning arbitrary grades (0–4) to the extent of damage based on the size/shape of comets, as opposed to a direct measurement of TL in µm, is an alternative way of quantifying DNA damage by the comet assay. This technique was used in four included studies, three of which demonstrate that the level of DNA damage was significantly higher in the IR-exposed compared to unexposed workers [51,58,78]. However, one study did not show any difference in DNA damage between 20 IR-exposed and 20 unexposed workers [66].

Gaetani et al. observed no difference between IR-exposed and unexposed workers for three types of DNA lesions: SBs, oxidized purines and oxidized pyrimidines assessed by the conventional and enzyme modified comet assay in peripheral blood cells [43].

A significant dose-response relationship was found between cumulative IR exposure dose and glycophorin A (GPA) mutation frequency in red blood cells of hospital workers, both for NO and NN variants (β = 1.88 × 10^−6^/cGy, *p* = 0.003; and β = 2.23 × 10^−6^/cGy, *p* = 0.0001, respectively) [46].

Using polymerase chain reaction (PCR), relative leukocyte telomere length (LTL) (as the ratio of telomere repeats to a single-copy gene, relative to a reference sample) was shown to decrease significantly with an increase in lifetime radiation dose (r = −0.319; *p* = 0.03), but also with an increase of occupational radiological risk score (r = −0.267; *p* = 0.002) [28]. The latter exposure parameter takes into account the number of years in catheterization laboratory, the number of procedures per year, and the distance from the source of radiation. Recently, using quantitative real-time PCR, a significantly higher rate of microdeletion and microduplication as assessed by copy number variation (CNV) in the SY1197 sequence-tagged site of the Y-chromosome azoospermia factor region c (AZFc) was shown in male Cath lab workers compared to unexposed controls (CNV = 1.53 ± 0.8 vs. CNV = 1.02 ± 0.4 respectively, *p* = 0.0005) [29]. However, a non-significant difference was shown for the SY579 site CNV in AZFc region between exposed and unexposed workers. A sequence-tagged site (STS) is relatively short (200–500 base pairs) and can be specifically amplified by PCR. In clinical setting, the STSs (such as SY579 and SY1197) have been used to detect microdeletions (and infertility) in Azoospermia (AZF) genes in men.

Lastly, the frequency of γ-H2AX foci, a marker of DNA double-strand breaks, was found to be significantly higher in IR-exposed workers compared to healthy volunteers [68].

## 4. Discussion

Potential genotoxic effect of medical occupational exposure to IR is a widely recognized concern that has been scrutinized in many studies in the last two decades. Although a great variety of genotoxicity endpoints has been assessed in these studies helping generate new knowledge, there are still inconsistencies in the results, making it difficult to interpret. In this systematic review and meta-analysis, we attempted to consolidate knowledge using commonly accepted methods with the overall objective to identify the biomarkers of genotoxicity that are most reliably and commonly observed at elevated levels in medical workers occupationally exposed to IR compared to unexposed cohorts. The approach implemented in our work produced a list of 65 studies (Table 2).

The results of our work confirm the relevance of CA and MN as genotoxicity biomarkers that are consistently elevated in IR-exposed vs. unexposed workers. SCE, stable translocations and the comet assay endpoints are strong candidates and require further studies to validate their usefulness.

Thus, the most commonly reported cytogenetic biomarkers were unstable CA (mainly dicentrics and acentric fragments) and MN, and both were significantly elevated in IR-exposed workers compared to controls in the majority of the studies (Table 3, Figure 2).

Our meta-analyses of the data from studies with complete information confirmed that IR-exposed medical workers had significantly elevated frequencies of blood lymphocytes with CA and/or MN. Particularly, dicentrics were previously reported to be “the biomarker of choice for investigating recent exposure to IR” which is typically little confounded by other factors [22] and is a standard endpoint for radiation biodosimetry applications [91]. Because of its unstable nature and continuous renewal of PBL, the frequency of dicentrics decreases with time after exposure. This may explain why decreases in unstable CA frequencies were found in workers upon removal from IR exposure [48]. Interestingly, such periods without IR exposure (vacation, break or change to non-IR professional activities) may have contributed to the failure to show dose-response relationships for unstable CA in nuclear medical workers [48,60]. However, regardless of the endpoint, establishing dose-response for genotoxicity biomarkers in human studies is a very challenging task due to several factors, such as uncertainty in dose estimates, type of IR, mode of exposure, time, etc.). Dose-responses for these endpoints are well established for controlled ex vivo irradiation of human PBL, but they poorly compare to the studies reviewed here in terms of lowest dose resolution. Indeed, most of the occupational exposures are below the lowest resolution power observed in ex vivo cytogenetic assays [92,93,94], but previous studies did show a significant increase in cytogenetic endpoints in patients who received radiation exposure during a single medical examination, such as a CT scan or catheterization procedure, where the radiation dose is far lower than 100 mSv [95,96,97]. Similarly the effect of confounding factors on CA frequencies was inconsistent between studies, with some authors reporting the effect of certain factors such as age, smoking and gender [54,57,60,72], while others showing no influence [62,64,76,87]. Furthermore, statistically significant differences in CA frequencies were reported between cohorts employed at different working places, with specificity of certain types of CA depending on the job. Translocations have not been extensively studied (6 studies out of the 30 studies dealing with CA), but showed a good consistency and reliability, with significantly higher values in IR-exposed workers in almost all studies. Unlike dicentrics, translocations are stable CA in PBL and can therefore be considered as retrospective biomarkers of exposures [14]. However, translocations were reported to be hypersensitive to other factors (e.g., age, smoking habits, mode of exposure, diet and exposure to other clastogenic agents) that can influence their accumulation and persistence [98].

Compared to CA, MN are much easier to score, either manually or using automated systems [14]. The MN assay can also be viewed as an alternative method to dicentric chromosome assay and presents the advantage that it can be assessed in lymphocytes (fresh or frozen), cell lines, erythrocytes, epithelial buccal cells, nasal mucosa or urine-derived cells using standardized protocols [99]. Thus, MN frequencies from epithelial buccal cells have recently been shown to be significantly higher in IR-exposed compared to unexposed health workers (studies not included in the present systematic review) [100,101]. The inconsistency between the sensitivity seen in ex vivo (20 mGy or higher) and in vivo studies (typically <20 mGy) for CA and dicentrics is also evident for the MN assay that is considered not to be very sensitive at low doses in ex vivo studies (~200 mGy lowest dose resolution) [93,102]. MN can be induced by other environmental agents suggesting MN is a non-specific biomarker of IR exposure [22]. Although the results suggest that MN is one of the best discriminators between IR-exposed and unexposed medicine workers (Figure 2, Figure 5), careful consideration of the named potentially confounding factors should be included in new studies, as well as in the analyses of published results.

SCE are easy to detect and score and were shown to be a sensitive and reliable endpoint of a genotoxic potential of chemical carcinogens and mutagens [103,104]. However, such sensitivity to chemical and relative insensitivity to IR [17] suggests that SCE measurements in low-dose IR-exposed humans should be carefully examined for potential co-exposure to chemical mutagens or other DNA damaging factors.

With regards to NPB frequencies, only three out of seven studies reported significantly higher NPB frequencies in IR-exposed medical workers compared to controls [33,58,65]. Furthermore, out of the three studies, Caradonna et al. reported extremely high values for NPB frequency compared to the rest of studies for this endpoint [33]. NPB are indicators of the presence of dicentric chromosomes and therefore their frequency are expected to be similar to that of dicentric chromosomes, which was not the case in this study. Additionally, the mean age in exposed and non-exposed groups in Caradonna et al. were different, 42 vs. 30 years old, and no adjustment was made in contrast to other studies. The overall mean difference between IR-exposed and unexposed workers was no longer significant when the study of Caradonna et al. was excluded (Figure S1). PCD were used in only two studies, which does not allow judging its usefulness as a biomarker in further studies. Lastly, ring chromosome frequencies were assessed in 14 studies, in which non-significant differences between IR-exposed and unexposed workers were found for most of the studies, suggesting that this endpoint can be excluded from the list of potential IR exposure biomarkers.

Molecular biomarkers, grouped here as DNA integrity endpoints, were dominated by the parameters that are measured using the comet assay. Thus, 12 out of 18 studies in this category used the comet assay to measure tail length, tail moment, %DNA in the tail or an arbitrary comet score or index, showing a good consistency and reliability, with significantly higher values in IR-exposed workers in most of the studies. It is not clear what parameter is the most relevant for genotoxicity assessment, and this has been the subject of debate because each comes with its advantages and limitations. It was first assumed that tail moment (product of %DNA and tail length) provides a better description of DNA integrity compared to tail length only [105]. However, tails with different lengths, numbers of fragments and relative amounts of DNA may have the same tail moment, which can be considered a counter-argument for the use of this descriptor [106]. An alternative way of scoring DNA damage by the comet assay, suitable for low budget and fast assessment, is a manual classification of comets into five categories based on their appearance [107]. Noteworthily, in the studies included in this review, these comet assay parameters were found to have little or no sensitivity to confounding factors such as gender, age, smoking status and alcohol consumption, suggesting potential specificity to IR exposure [52,59,71]. Nevertheless, it can be noted that differences in TL were found between various hospital departments and working places; however, no common pattern across studies was seen [44,52,59,82]. It should be pointed that the types of DNA lesions detected by the comet assay (SSB, oxidized and alkali-labile sites) are highly abundant in cells due to oxygen metabolism and replication which has to be considered while interpreting results. These lesions are rapidly repaired after irradiation (minutes to hours) and thus should not be interpreted as the actual DNA damage induced directly by protracted low-dose IR exposure [108]. Instead, the detected increases in DNA damage levels in lymphocytes of IR-exposed subjects may reflect secondary effects due to altered repair machinery, genomic instability or additional production of reactive oxygen species. Moreover, one of the main limitations of the comet assay is the inter-laboratory variability in protocols, affecting results and, subsequently, the comparability between studies.

Glycophorin A (GPA) mutant, leukocyte telomere length, copy number variation in AZFc region, and γH2AX foci were assessed only in one study for each endpoint, and were found to be elevated in IR-exposed cohorts. However, no correlation between age or duration of occupational exposure and γ-H2AX foci frequency in IR-exposed medical workers were seen. Each of these endpoints is relevant to a very specific type of DNA lesions and, when examined separately, may be poor indicators of the overall burden of genotoxicity.

The biomarkers included in this work have been widely investigated by previous molecular epidemiology studies to assess environmental, occupational and medical exposure to IR. Although there are still uncertainties with respect to their sensitivity or specificity to detect low level of IR exposures in human biomonitoring [7], it is worth considering these important aspects in the interpretation of the results. To this end, Table 4 shows a summary of the dose detection limit and specificity for each biomarker used in our study.

Relevance to an adverse health outcome is an important attribute of a genotoxicity biomarker since inferring a potential health risk is one of the main objectives of measuring such a biomarker in IR exposed individuals. The greater relevance, the higher value of such assessment. Although high frequencies of CA in PBL have long been linked to an increased risk of cancer using association [11,12,13,109] and incidental evidence [110], the causal relationship between CA in PBL and cancer has not been established. Recent results of the analysis of genomic characteristics of thyroid cancer in IR-exposed vs. unexposed patients (I-131 from Chernobyl nuclear accident) suggest that radiation exposure was associated with increased frequencies of small insertions/deletions and other small structural chromosome variants originating from DSB [9]. Although indirectly, these results highlight the importance of CA in tumorigenesis and thus their value as a genotoxicity biomarker in nuclear medicine workers.

Like unstable CA, MN represent a lethal abnormality that typically results in cell death during mitosis, thus preventing potential neoplastic transformation of the cell and suggesting a poor link to cancer risk. However, recent findings suggest that MN may not be passive outcomes of earlier DNA damage events, but exert biological activity triggering hypermutation and pro-inflammatory signaling [111,112]. Since lymphocytes are known to actively penetrate tissues, it is feasible to hypothesize that pro-inflammatory signaling by micronucleated lymphocytes may contribute to chronic tissue inflammation which in turn would increase the risk of tumorigenesis [113]. Together with these recent highlights on the role of MN in mutagenesis and tissue homeostasis, our results confirming that MN are reliably detected at elevated rates in PBL of IR-exposed medical workers highlight the potential value of this biomarker.

Unfortunately, our systematic review showed a limited number of studies assessing dose-responses, resulting in inability to carry out quantitative dose-response meta-analysis. Furthermore, given that non-significant results are less likely to be reported [114], it can be assumed that the dose–response relationship between occupational medical IR exposure and cancer biomarkers is poorly understood [27,64,71,74]. Issues associated with dose records include for example underestimation of dose when personal badges are not properly worn as observed in cardiologists studies [115], which could affect corresponding dose-response estimates [116]. However, IR-exposure assessed by the duration of employment as proxy did not show more conclusive results [24,30,72]. Biological factors that can affect the shape of a dose-response for genotoxicity endpoints include a large variety of adaptive stress responses that can be induced at low doses of IR and used by cells to effectively eliminate genotoxic damage, predominantly by activation of DNA repair [117,118,119]. Low-dose IR was also shown to trigger anti-inflammatory processes [120,121] and immune activation [122,123], both capable of affecting the shape of the dose-response. The decrease in CA frequencies found in medical workers after vacation or removal from IR exposure [48,60] are consistent with these compensatory biological mechanisms. Interestingly, an alternative explanation of these results could be a withdrawal from the chronic work-/life-related stress also known to cause accumulation of DNA and chromosomal damage [124,125]. Indeed, chronic stress is known to cause DNA damage [126]. Similarly, exam-associated stress can lead to increased DNA damage in university students [127] and stress-relieving hormonal therapy was associated with a marked reduction of cancer risk [128]. Therefore, in complex real-life situation studies such as those carried out on nuclear medical workers, it is very important to consider a multitude of variables and factors. These and other factors discussed above that can potentially affect the results of the assessment of genotoxicity biomarkers may have contributed to high heterogeneity revealed by our meta-analyses. However, we did not examine the interaction effect between IR and potentially confounding factors that—as shown above for many endpoints—can influence the results. In fact, the number of studies where the endpoints were measured separately in populations stratified by other factors (e.g., in smoker vs. non-smokers) was very limited, preventing the interaction effect tests. Such analyses are arguably very important in future studies and meta-analyses to understand the causal relationship between a genotoxic endpoint and IR exposure.

Further, a complex dependence of the endpoints measured on a type of a medical department of employment or occupation was reported, certainly because of the type of radiation and the distance with the source [37,52,67], constituting another potential factor of data heterogeneity. A limited number of studies stratifying by department/occupation made it impossible to account for this information and/or detect a pattern.

Genetic susceptibility may account for inter-individual differences in radiation sensitivity [27,30,61,71,82] and further contribute to data variability. Specifically, the genetic polymorphism of DNA repair and xenobiotic-metabolizing enzymes may play a crucial role in determining an individual’s ability to repair cellular DNA after IR exposure, and therefore, to influence the biological endpoint and the dose-response relationship [27,30,61,71,82,129]. Progress in understanding the interaction of the genotype and genotoxic insults, such as IR, and how it affects cancer risks may pave the way towards future personalized radiation protection principles and approaches.

To our knowledge, this work is the first systematic review and meta-analysis of literature assessing genotoxicity biomarkers in medical workers exposed occupationally to IR. We included a broad range of endpoints ranging from molecular DNA events to cytogenetic rearrangement, resulting in a large number of studies covered. All included studies met the previously defined criteria according to the PRISMA recommendations, allowing robust and exhaustive analysis while maintaining focus on the main research question. In spite of all the revealed limitations of the reviewed studies, which is partially due to a broad range of covered genotoxicity endpoints, using the combination of both qualitative and quantitative descriptions, we were able to provide an overview of the status-quo in the area of genotoxicity biomarkers in healthcare professionals occupationally exposed to IR. Although the quality of the various analyses used for the measurement of genotoxicity in individual studies was not assessed (e.g., see such assessment in [130]), we applied the Newcastle–Ottawa Scale to assess the quality of the included studies, resulting in the exclusion of only one study with a score <3, thus underlining the good overall level of the considered studies.

Finally, in an attempt to account for confounding factors potentially correlated with certain endpoints, most studies did match the IR-exposed and unexposed groups by age and gender (and sometimes by smoking habits) and reported crude values, which we used in our analyses. This made it possible to handle confusion bias in our work.

## 5. Conclusions

We reviewed all available data on genotoxicity biomarkers in health workers exposed to IR occupationally using systematic review and meta-analyses. Our qualitative and quantitative results suggest that CA (mainly dicentrics and acentric fragments) and MN are the best discriminators and correctly reflect the interaction between the biological system of healthcare workers and low-dose IR exposure. In contrast, ring chromosomes and nucleoplasmic bridges appear to correlate poorly with medical occupational IR exposure and can be excluded from the list of potential IR exposure biomarkers. Among the DNA integrity biomarkers, the comet assay endpoints showed good correlation with IR exposure, however, it is DNA DSB, not SSB or single nucleotide variants that are thought to contribute to IR-induced cancer. Thus, the relevance of the comet assay results to long-term health conditions is unclear. Several factors that can contribute to the measured value of a genotoxicity marker exist (increasing variability and uncertainty of results) and should be better accounted for in future work; these include time away from IR exposure, repair mechanisms, age and life style. Other new biomarkers and techniques, such as telomere length as well as gene array techniques, may be highly useful to improve overall biological understanding of low dose radiation exposure and the likelihood of subsequent disease as well as to identify underlying factors that modulate radiation sensitivity.

Lastly, our review revealed a shortage of studies with accurate dosimetric information, thus emphasizing the need for dose evaluation to facilitate the construction of dose-responses. Our results warrant and inform future studies aiming at examining the role of specific types of CA and MN in long-term health outcomes, with prospective epidemiological studies of proper design being instrumental for achieving this daunting goal.

## Figures and Tables

**Table 1:** Selection criteria (PECO statements).

**PECO elements**	**PECO Question Formulation:** *What genotoxicity biomarkers can be used in future similar reviews or future prospective epidemiological studies to examine their association with long-term health outcomes following IR-exposure?*
**Population**	All studies involving medical workers, regardless of the profession and service
**Exposure**	Studies dealing with ionizing radiation from medical sources and containing dose estimates or surrogates
**Comparator**	− Comparison between exposed and unexposed workers − Comparison between different categories of exposure − Studies with a dose-response relationship or risk estimates
**Outcome**	Frequencies of micronuclei or chromosome aberrations or sister chromatid exchanges, telomere length and DNA damage parameters

**Table 2:** List and characteristics of the included studies.

Study	Country Sample Size Design	Healthcare Services, Department, Units, Occupations of Hospital Workers Exposed to IR	Exposure Assessment (Mean of Exposure) ^a^ and/or Type of Exposure	Biomarker(s)	Major Results	NOS Score
Andreassi et al. (2009) [27]	Italy N = 77 40 exposed/ 37 unexposed	Cardiac catheterization laboratories (interventional cardiologists)	Badge doses (1.6 ± 2.4 mSv for the last 6 months) DOE to IR (12.0 ± 9.9 years)	MN	19.7 ± 7.8 (E); 13.5 ± 6.3 (NE) R: 0.265 β = 0.34 (*p* = 0.004)	6
Andreassi et al. (2015) [28]	Italy N = 445 223 exposed/ 222 unexposed	Catheterization laboratories	Lifetime cumulative professional exposure reconstruction (21.1 ± 26.3 mSv) DOE to IR (12.2 ± 8.3 years) ORRS (18.5 ± 20)	Leukocyte telomere length	R: −0.319 β = −0.14 (*p* = 0.03)	6
Andreassi et al. (2020) [29]	Italy N = 130 83 exposed/ 47 unexposed	Catheterization laboratories (Cath lab workers)	DOE to IR (median = 6 years (IQR 1–25)) ORRS (median = 11 (IQR = 1–63))	Copy number status (microdeletion and microduplication) in AZFc region for two markers	OR_adjusted_ (SY1197) = 2.66 (95% CI: 1.09–6.31), *p* = 0.02	6
Angelini et al. (2005) [30]	Germany N = 42 21 exposed/ 21 unexposed	Units of Radiology Radiotherapy Cardiology (physicians and technicians)	Badge doses (40.6 ± 37.7 mSv)	MN	MN: 8.6 ± 2.8 (E); 6.7 ± 2.7 (NE) β = 0.004 (*p* = 0.941)	6
Bhatti et al. (2007) [31]	USA N = 152 Cohort	Radiologic technologists who began working before 1950 (USRT study)	Estimated cumulative occupational red bone marrow radiation dose score (1.9 ± 1.4 cGy)	FISH for translocations	ERR = 0.09/100 CE per mGy (95% CI −0.01–0.20, *p* = 0.07)	7
Bouraoui et al. (2013) [32]	Tunisia N = 110 67 exposed/ 43 unexposed	Nuclear medicine Radiology Orthopedic Radiotherapy Physiology Cardiology departments	DOE to IR (18.4 ± 9.3 years) X-ray, γ-ray,^125^I, ^131^I, ^57^CO, etc.	MN	13.6 ± 4.9 (E); 6.5 ± 4.2 (NE) β = 0.7 (*p* = 0.04)	6
Caradonna (2015) [33]	Italy N = 110 56 exposed/ 54 unexposed	(clinicians, technicians, attendants)	-	NPB CA (Chromatid breaks, chromosomal breaks, dicentrics, radial configurations)	CA: 2.87 ± 0.17 (E); 1.15 ± 0.05 (NE) NPB: 5.18 ± 0.23 (E); 1.42 ± 0.15 (NE)	3
Cigarran et al. (2001) [34]	Spain N = 38 20 exposed 18 unexposed		Badge doses (38.1 ± 31.7 mSv)	CA (translocations, dicentrics)	Translocations: 1.04 ± 0.11 (E); 0.90 ± 0.12 (NE) Dicentrics: 0.09 ± 0.03 (E); 0.15 ± 0.04 (NE)	4
Dias et al. (2007) [35]	Brazil N = 72 36 exposed/ 36 unexposed	Radiology Units (physicians, technicians)	DOE to IR (6.5 ± 5.0 years)	CA (chromatid breaks, chromosome breaks, exchange figure) MN	MN: 6.13 ± 3.18 (E); 5.11 ± 3.85 (NE) CA: 2.60 ± 2.00 (E); 2.30 ± 1.80 (NE)	5
Djokovic et al. (2016) [36]	Serbia N = 65 Cohort	Nuclear Medicine Centre	Badge doses	CA (dicentrics, acentrics, rings, chromatid lesions, isochromatid lesions)	No significant difference for dicentrics, rings, chromatid lesions between the initial and periodical medical examinations (during exposure), but significant increase of acentric fragments	6
Dobrzynska et al. (2014) [37]	Poland N = 86 46 exposed/ 40 unexposed	Nuclear Medicine Oncological Endocrinology (doctors, nurses, technicians, radiochemists and administrative staff)	Badge doses (0.3 ± 0.2 mSv/year) DOE to IR (8.5 ± 6.7 years)	TM % DNA	TM: 0.90 ± 1.09 (E); 0.30 ± 0.44 (NE) %DNA: 1.60 ± 1.50 (E); 0.78 ± 0.54 (NE)	6
Doukali et al. (2017) [38]	Tunisia N = 64 29 exposed/ 35 unexposed	Radiotherapy Radiology departments	DOE to IR (8.8 ± 4.1 years in Group I, 20.1 ± 4.7 years in Group II)	MN SCE	MN: 1.16 ± 0.65 (E); 0.46 ± 0.21 (NE) SCE: 8.47 ± 0.45 (E); 7.22 ± 0.82 (NE)	4
Eken et al. (2010) [39]	Turkey N = 70 40 exposed/ 30 unexposed	Radiology unit (physicians, technicians)	Badge doses (median = 0.17 (range 0.10–3.86 in the last 6 months)	MN SCE	MN: 6.88 ± 2.54 (E); 5.50 ± 2.00 (NE) SCE: 6.86 ± 0.44 (E); 6.70 ± 0.53 (NE)	7
El-Benhawy et al. (2016) [40]	Egypt N = 90 60 exposed/ 30 unexposed	Radiotherapy Diagnostic radiology Industrial radiographers	Badge doses (2.9 ± 1.9 mSv/year in radiologists, 3.1 ± 1.5 mSv/year in radiotherapists)	CA (gaps, breaks, fragments and dicentrics)	All types of CA in (E) significantly higher than in (NE)	6
Engin et al. (2005) [41]	Turkey N = 75 20 + 33 exposed/ 22 unexposed	Radiotherapy Radio-diagnostic	Badge doses DOE to IR (11.2 ± 0.8 years in X-ray group, 6.5 ± 0.9 in γ-rays group) γ-rays and X-rays	SCE	10.50 ± 0.41 (E); 4.17 ± 0.32 (NE)	4
Fang et al. (2019) [42]	China N = 334 175 exposed/ 159 unexposed		Badge doses (38.4 ± 27.4 mSv) X-ray radiation	CA (dicentrics, ring, and acentric fragments) MN % DNA TM & Olive TM	MN, CA, %DNA, TM significantly greater for (E) compared to (NE)	8
Gaetani et al. (2018) [43]	Italia N = 248 116 exposed/ 132 unexposed	Department of Nuclear Medicine Radiology Interventional Radiology Oncological Radiotherapy (doctors, nurses, technicians and radiochemists)	Badge doses (1.9 ± 1.6 mSv in group with accumulated IR dose <6 mSv; 34.0 ± 30.4 in group with accumulated IR dose >6 mSv)	DNA SBs	No difference in SBs frequencies between IR dose groups	6
Gao et al. (2020) [23]	China N = 336 218 exposed/ 118 unexposed	Diagnostic radiology Radiotherapy Interventional radiology Nuclear medicine (technicians, physicians and nurses)	Badge doses (median = 0.5 mSv (IQR = 0.4–0.7))	MN	MN (median, IQR): 3 (1, 5) (E); 2 (0.75, 4) (NE))	7
Garaj-Vrhovac et al. (2003) [44]	Croatia N = 100 50 exposed/ 50 unexposed	Radiology Surgery	Badge doses (range 0–8548 μSv in the previous year)	TL TM	TL: 14.85 ± 0.21 (E); 11.46 ± 0.15 (NE) TM: 17.49 ± 0.23 (E); 14.05 ± 0.13 (NE)	5
Gerić et al. (2019) [45]	Croatia N = 48 24 exposed/ 24 unexposed		Badge doses (1.8 ± 3.6 mSv over the last year) X-rays	MN NPB TL %DNA	MN: 5.74 ± 3.89 (E); 5.38 ± 2.63 (NE) NPB: 1.61 ± 1.08 (E); 1.38 ± 1.65 (NE) TL: 15.46 ± 1.47 (E); 14.05 ± 1.36 (NE) %DNA: 1.57 ± 0.47 (E); 1.49 ± 0.89 (NE)	6
Gharibdousty et al. (2017) [24]	Iran N = 70 35 exposed/ 35 unexposed	(Radiopharmacists)	Badge doses (6.6 ± 5.8 mSv in the last year)	MN NPB	MN: 25.82 ± 8.67 (E); 10.52 ± 6.83 (NE) NPB: 1.02 ± 0.02 (E); 0.85 ± 0.37 (NE)	6
Ha et al. (2002) [46]	Korea N = 176 Cross sectional	144 workers in two nuclear power plants 32 workers in one university hospital	Badge doses (0.9 ± 1.3 cGy for hospital workers)	Glycophorin A mutant assay (NO or NN variants)	NO variant: β = 1.88 (*p* = 0.003) NN variant: β = 2.23 (*p* = 0.0001)	3
Joseph et al. (2004) [47]	India N = 73 46 exposed/ 27 unexposed	Nuclear Medicine	Badge doses (range 0.25–62.9 mSv)	MN	9.80 ± 6.20 (E); 7.00 ± 3.80 (NE)	6
Jovicic et al. (2009) [48]	Serbia N = 94 30 exposed/ 64 unexposed		Badge doses DOE to IR (years) X-ray	CA (chromatid and chromosome breaks, acentrics, dicentrics and rings)	Aberrant cells: 3.40 ± 1.80 (E); 0.80 ± 0.90 (NE)	6
Jovicic et al. (2010) [49]	Serbia N = 53 30 exposed/ 23 unexposed		Badge doses (13.3 mSv (range 4.81−24.76)) DOE to IR (12.7 ± 7.4 years) X-rays	CA (chromatid and chromosome breaks, acentrics, dicentrics and rings) PCD	CA and PCD significantly higher in (E) compared to (NE) (except rings) R Total life effective dose-PCD = 0.71 (*p* < 0.001)	6
Kasuba et al. (2008) [50]	Croatia N = 785 765 exposed/ 200 unexposed	Anesthesiologists, anesthetic technicians, radiology technicians, operating room nurses, surgeons, nurses, radiologists, and urologists/gynecologists	DOE to IR (12.1 ± 8.40 to 15.8 ± 9.8 years)	CA (dicentrics and rings, acentric fragments, and tri- and tetra-radial exchanges)	CA significantly higher in (E) compared to (NE) (except rings)	5
Khisroon et al. (2015) [51]	Pakistan N = 144 74 exposed/ 70 unexposed	Radiology personnel	DOE to IR (7.8 ± 5.3 years)	CS	CS: 129.8 ± 17.2 (E); 53.0 ± 25.0 (NE) R DOE-CS = 0.62 (*p* < 0.001)	6
Kopjar et al. (2005) [52]	Croatia N = 120 60 exposed/ 60 unexposed	Nuclear medicine physicians, technical experts, engineers, nurses, cleaners	Badge dose (196 µSv (range 0–1401) Radionuclides (dominantly ^131^I and 99mtc)	TL CA (number of sister chromatids and breakage events)	TL: 21.44 ± 0.14μm (E); 13.96 ± 0.02μm (NE) CA mean: 2.37 ± 0.16 (E); 0.85 ± 0.09 (NE) Aberrant cells: 1.15 ± 0.08; 0.23 ± 0.06 (NE)	6
Kumar et al. (2016) [53]	India N = 134 83 exposed/ 51 unexposed	Diagnostic or therapeutic radiation facilities	Badge doses DOE to IR (6.5 ± 0.7 years)	MN CA (aneuploidy, acentric fragments)	CA and MN frequencies significantly higher in (E) compared to (NE)	5
Lalic et al. (2001) [54]	Croatia N = 45 25 IR-exposed/ 20 non-IR exposed	(Medical radiology, X-rays technicians, nurses)	Badge doses	CA (chromatid and chromosome breaks, acentric fragments, dicentrics)	Total number of CA: 4.08 ± 0.37 (E); 4.35 ± 0.50 (NIR) R with 6-years exposure dose = 0.62	4
Little et al. (2014) [55]	USA N = 238 Cohort	USRT study CTS-I: inclusions in 1994–1995 CTS-II: inclusions in 2003	Estimated cumulative personal-diagnostic-medical Occupational red bone marrow radiation dose scores	FISH for translocations	Translocation rates in relationship to occupational and personal-diagnostic-medical (PDM) doses = 7.0 (95% CI 1.2, 12.9) × 10^−2^ translocations Gy^−1^	7
Maffei et al. (2002) [56]	Italy N = 74 37 exposed/ 37 unexposed	(Physicians and technicians)	Badge doses (35.1 ± 40.8 mSv) X and γ-rays	MN	MN: 6.78 ± 4.92 (E); 5.54 ± 2.99 (NE)	6
Maffei et al. (2004) [57]	Italy N = 69 34 exposed/ 35 unexposed	(Physicians and technicians)	Badge doses (35.8 ± 38.9 mSv) X and γ-rays IR-exposure for at least 3 years	CA (chromatid breaks, chromatid exchanges, chromosome breaks and chromosome exchanges)	Aberrant cells: 2.87 ± 3.10 (E); 1.08 ± 1.03 (NE)	6
Maluf et al. (2001) [58]	Brazil N = 44 22 exposed/ 22 unexposed		Badge doses (range 0.2–121.8 mSv) X-rays	MN NPB CS	MN: 8.84 ± 2.35 (E); 7.18 ± 2.59 (NE) NPB: 2.98 ± 1.57 (E); 1.96 ± 1.04 (NE) CS: 17.73 ± 10.51 (E); 8.54 ± 7.11 (NE)	6
Martínez et al. (2010) [59]	Mexico N = 61 41 exposed/ 20 unexposed	Nuclear Medicine Radiotherapy Radiology	Badge doses (0.21 ± 0.02 mSv/month, 0.4 ± 0.2 mSv/month, 0.17 ± 0.02 in Nuclear Medicine, radiotherapy, and radiology departments respectively)	TL	Radiology: 28.6 ± 3.50 (E); 15.2 ± 1.92 (NE) Nuclear: 92.5 ± 19.02 (E); 15.2 ± 1.92 (NE) Radiotherapy: 63.4 ± 15.4 (E); 15.2 ± 1.92 (NE)	4
Milacic et al. (2005) [60]	Serbia and Montenegro N = 68 46 exposed/ 22 unexposed		Badge doses (7.9 ± 5.0 mSv) DOE to IR (10.6 ± 6.4 years) X-rays IR-exposure for at least 3 years	CA (dicentrics, rings and acentric fragment, breaks, exchanges)	CA frequencies correlate with absorbed doses. During breaks of exposure, number of damaged cells decreased Time necessary for aberrations to disappear not in relation with former frequency of aberrations or DOE and absorbed dose	5
Milic et al. (2015) [61]	Croatia N = 147 77 exposed/ 70 unexposed		DOE to IR (13.7 ± 8.9 years)	MN NPB	MN: 16.20 ± 10.40 (E); 11.50 ± 9.40 (NE) β = 0.403 (*p* = 0.003) NPB: 0.90 ± 1.50 (E); 1.70 ± 4.00 (NE) β = 0.024 (*p* = 0.230)	6
Movafagh et al. (2007) [62]	Iran N = 93 50 exposed/ 43 unexposed	Radiotherapy	Badge doses X-rays IR-exposure for at least 5 years	CA (Dicentrics, Fragments and Rings)	Total CA: 3.40 ± 1.18 (E); 2.00 ± 0.82 (NE)	6
Mrdjanovic et al. (2005) [63]	Serbia-Montenegro N = 45 30 exposed/ 15 unexposed	Radiotherapy Cardiology	DOE to IR (11.9 ± 9.04 years)	SCE MN	MN for Radiology group: 15.00 ± 9.39 (E); 9.06 ± 3.23 (NE) SCE: no significant difference between (E) and (NE)	5
Pajic et al. (2016) [64]	Serbia N = 90 50 exposed/ 40 unexposed		Badge doses (9.9 ± 6.8 mSv in last 5 years) DOE to IR (18.0 ± 8.1 years) Radionuclides (Y^90^ and I^131^)	CA (chromatid and isochromatid breaks, acentrics, dicentrics and rings) MN PCD	MN (ratio per number of analyzed cells): 1/48.26 (E); 1/117.3 (NE) Dicentrics (ratio): 1/1600 (E); 1/303.03 (NE) Acentrics (ratio): 1/533 (E); 1/75.75 (NE) Chromatid breaks (ratio): 1/615 (E); 1/294 (NE) Isochromatid breaks (ratio): 1/1143 (E); 1/400 (NE) PCD (ratio): 1/800 (E); 1/94.33 (NE) R for DOE-aberrant cells = 0.77 R for DOE-MN = 0.82 R for DOE-PCD = 0.65	5
Pajic et al. (2017) [65]	Serbia N = 402 201 exposed/ 201 unexposed	Radiology	DOE to IR (15.1 ± 7.4 years) X-rays	MN NPB	MN: 15.15 ± 5.82 (E); 8.31 ± 3.88 (NE) NPB: 0.75 ± 0.85 (E); 0.23 ± 0.47 (NE)	7
Pakniat et al. (2016) [66]	Iran N = 40 20 exposed/ 20 unexposed	Radiology CT scan	Badge doses	CS	No significant difference between (E) and (NE) at baseline After irradiation by 4mGy, DNA damage frequencies significantly lower in (E) compared to (NE)	4
Qian et al. (2016) [67]	China N = 1535 1392 exposed/ 143 unexposed	Radiodiagnostic Radiotherapy	Badge doses (13.7 mSv (range 0.2–19.8))	MN CA (dicentric, centric ring, and acentric fragment, translocation, inversion, insertion, and deletion) with FISH	Frequencies of CA and MN rates in (E) significantly higher than (NE) (0.68 vs. 0.22%, and 2.44 vs. 1.72‰ respectively)	6
Raavi et al. (2016) [68]	India N = 150 20 exposed/ 130 unexposed	Radiology (physicians, staff)	Badge doses (range 0.02–0.40)	γ-H2AX foci	Mean γ-H2AX foci: 0.066 ± 0.005 (E); 0.042 ± 0.001 (NE)	5
Ropolo et al. (2012) [69]	Italy N = 60 30 exposed/ 30 unexposed		Badge doses (19.5 ± 37.59 mSv) DOE to IR (12.5 ± 9.5 years) X- and gamma-radiation	MN NPB	MN: 3.87 ± 2.14 (E); 3.66 ± 1.68 (NE) NPB (median (range)): 0.50 (0–2.75) (E); 0.75 (0–2.25) (NE)	5
Sahin et al. (2009) [70]	Turkey N = 21 “auto-controls”	Nuclear medicine	Badge doses (4.0 ± 10.2 mSv in last year) Occupational radiation exposure between two vacations and after 1 month of vacation either following or before occupational exposure	MN SCE	MN: 21.90 ± 1.71 (AE); 14.13 ± 1.25 (BE) SCE: 7.52 ± 0.27 (AE); 6.25 ± 0.17 (BE)	5
Sakly et al. (2012) [71]	Tunisia N = 64 31 exposed/ 33 unexposed	Radiology	DOE to IR (13.7 ± 9.4 years)	TL	24.98 ± 1.07 µm (E); 22.44 ± 0.57 µm (NE)	6
Sakly et al. (2013) [72]	Tunisia N = 87 60 exposed/ 27 unexposed	Radiology Cardiology	DOE to IR (16.5 ± 10.2 years, 12.3 ± 9.4 years in radiology and cardiology departments respectively)	MN CA (gaps, simple-strand breaks and double-strand breaks, reciprocal translocations, rings, and dicentrics)	MN in Radiology: 21.90 ± 4.23 (E); 10.78 ± 1.47 (NE) MN in Cardiology: 25.57 ± 4.79 (E); 10.78 ± 1.47 (NE) CA in Radiology: 33.63 ± 4.40 (E); 14.26 ± 3.40 (NE) CA in Cardiology: 35.37 ± 5.19 (E); 14.26 ± 3.40 (NE)	5
Santovito et al. (2014) [73]	Italy N = 42 21 exposed/ 21 unexposed	Radiology	-	CA (chromatid breaks, chromosome breaks, dicentrics, acentric fragments, and Tri- or Tetra-radials, Gaps) SCE	Aberrant cells: 2.07 ± 0.17 (E); 1.17 ± 0.17 (NE) β = −0.08 (95% CI −2.22;3.76) per years of employment SCE: 6.67 ± 0.29 (E); 4.49 ± 0.39 (NE) β = 0.26 (95% CI −8.07;10.32) per years of employment	5
Sari-Minodier et al. (2007) [74]	France N = 201 132 exposed/ 69 controls	Radiotherapy Nuclear medicine Cardiology Radiology Pediatric operating room	Badge doses (0.17 ± 0.47 mSv in the last year) + Estimated medical radiation dose as a patient	MN	14.90 ± 8.10 (E); 11.80 ± 6.50 (NE) β = 2.55 (95% CI 0.57;4.53, *p* = 0.012) (increase for (E) people vs. (NE))	6
Scarpato et al. (2006) [75]	Italy N = 92 Cross-sectional	Orthopedic Radiology Cardiology	Badge doses (3.3 ± 5.6 mSv in the last 3 years) IR-exposure for at least 3 years	CA (breaks or fragments, quadri-radial and triradial, translocations, dicentrics and rings)	Total CA: 1.79 ± 0.23 (HE); 1.37 ± 0.24 (ME); 1.32 ± 0.19 (LE)	5
Shafiee et al. (2020) [76]	Iran N = 81 46 exposed/ 35 unexposed	Lithotripsy CT scan Digital radiology	Badge doses (range 0–2.99 mSv in the last year)	MN CA (acentric fragments, gap, rings, and dicentrics)	MN: 6.89 ± 2.25 (E); 5.17 ± 1.70 (NE) R with cumulative radiation dose = 0.98 (*p* = 0.02) Significantly higher frequencies of CA in (E) compared to (NE) (except dicentrics and rings). R with cumulative radiation dose = 0.97 (*p* = 0.02)	6
Siama et al. (2019) [77]	India N = 66 33 exposed/ 33 unexposed	Radiology	Badge doses (40.9 ± 39.9 mSv) DOE to IR (10.3 ± 7.1 years)	MN	Significant rise in MN frequency in (E) compared to (NE) β = 0.42 (*p* = 0.02) per years of employment	6
Silva et al. (2016) [78]	Brazil N = 90 45 exposed/ 45 unexposed	(Radiologists, technologists and technicians)	X-rays	CS	Significantly higher damages (minimum to maximum levels) in (E) compared to (NE) Significantly lower cells with no damage in (E) compared to (NE) R with time of work = 0.637 (*p* = 0.001)	5
Surniyantoro et al. (2018) [79]	Indonesia N = 101 81 exposed/ 20 unexposed	Radiology Radiotherapy (doctors, radiologists, radiotherapists, and nurses)	Badge doses (0.2 ± 0.2 mSv/year) DOE to IR (20.8 ± 7.5 years)	MN	15.38 ± 7.72 (E); 9.00 ± 5.49 (NE) β = 0.05 (*p* = 0.69)	5
Thierens et al. (2000) [80]	Belgium N = 131 71 exposed/ 60 unexposed	Radiology Radiotherapy Nuclear Medicine Cardiology Urology Gastroenterology (doctors, nurses or technicians)	Badge doses (20.8 ± 7.5 mSv)	MN	21.88 ± 13.46 (E); 18.36 ± 7.53 (NE)	6
Tug et al. (2013) [81]	Turkey N = 74 39 exposed/ 35 unexposed	(Radiology technologists)	-	SCE	5.19 ± 1.06 (E); 3.38 ± 1.13 (NE)	4
Vellingiri et al. (2014) [82]	India N = 112 56 exposed/ 56 unexposed	Radiology Cardiology Orthopedic (nurses, technicians, physicians)	Badge doses (range 1.3–24.5 mSv) DOE to IR (years)	CA (dicentrics or unusual karyotypes and structural CA) MN TL TM	Significantly higher CA and MN frequencies, TL and TM in (E) compared to (NE)	7
Vral et al. (2016) [83]	Belgium N = 38 29 exposed/ 19 unexposed	Nuclear medicine Interventional radiation	Badge doses (Hp(10) 4.95 ± 2.00 mSv over the last year in Nuclear Medicine department)	MN	No significant difference between (E) and (NE)	6
Wang et al. (2017) [84]	Japan N = 530 Cross sectional		Badge doses (means from 0.4 to 1.7 mSv/year)	CA (Chromosome breaks, fragments and dicentrics) MN	No significant difference in CA and MN between years of service groups, except significantly higher CA in female with >20 years compared to lower classes	4
Zakeri et al. (2003) [85]	Iran N = 508 450 exposed/ 58 unexposed	(Industrial radiographers, nuclear research center, nuclear medicine workers, medical X-ray diagnostic workers)	-	CA (dicentrics, rings and acentrics)	Acentrics and dicentrics significantly higher in the different job-groups of (E) compared to (NE)	5
Zakeri et al. (2004) [86]	Iran N = 107 71 exposed/ 36 unexposed	Cardiovascular laboratory (cardiologist, nurses and technicians)	Badge doses (range 0.25–15 mSv/year) DOE to IR (11 ± 7 years) X-rays	CA (dicentrics, and acentrics) MN	MN: 38.91 ± 15.58 (E); 11.05 ± 4.51 (NE) CA: 6.73 ± 2.23 (E); 1.0 ± 0.5 (NE)	6
Zakeri et al. (2010) [87]	Iran N = 136 101 exposed/ 35 unexposed	(Interventional cardiologist, nuclear medicine physicians, conventional radiologists)	Badges doses (range 0.25–48 during the previous year)	CA (gap, isogap, break, minute, fragment, dicentric) MN	MN: 21.5 ± 9.6 (IC); 19.7 ± 3.8 (NM); 16.8 ± 8.1 (CR); 11.8 ± 6.5 (NE) %acentrics: 3.23 ± 2.60 (IC); 2.87 ± 1.40 (NM); 2.18 ± 0.90 (CR); 1.28 ± 0.50 (NE) %dicentrics: 0.21 (IC); 0.14 (NM); 0.13 (CR); 0.04 (NE)	6
Zakeri et al. (2010) [88]	Iran N = 74 37 exposed/ 37 unexposed	(Interventional cardiologists, clinical physicians)	Badges doses (8.1 ± 7.8 mSv/year; 30.5 ± 24.3 over the last 5 years)	CA (Chromatid and chromosome breaks, gaps, dicentrics and centric rings)	Aberrant cells: 2.78 ± 1.63 (E); 1.27 ± 1.07 (NE)	5
Zhou et al. (2016) [89]	China N = 127 52 exposed/ 75 unexposed	Radiology Cardiology (radiologic technologist, radiologist, and interventional cardiologist) Participants with cataract	DOE to IR (9.3 ± 2.8 years)	CA (dicentrics, tricentrics, structural)	1.77 ± 0.92 (E); 0.63 ± 0.51 (NE)	4

^a^ Mean ± S.D., when available; (AE) after exposure; (BE) before exposure; (E) IR-exposed medical workers; (HE) high exposure; (LE) low exposure; (ME) medium exposure; (NE) unexposed workers; (NIR) non-IR-exposed workers; (CR) conventional radiologist; (IC) interventional cardiologist; (NM) nuclear medicine physician; (%DNA) %DNA in the tail; (CA) chromosome aberrations; (CS) comet score; (DOE) duration of occupational exposure; (ERR) excess relative risk; (MN) micronucleus; (NPB) nucleoplasmic bridges; (ORRS) occupational radiological risk score; (PCD) premature centromere division; (R) correlation; (SBs) stand breaks; (TL) tail length; (TM) tail moment.

**Table 3:** Summary of the biomarkers used in the included studies.

Endpoints	Number of Studies Carried Out on These Endpoints	Is This Biomarker Recommended for Use in Future Prospective Epidemiological Studies to Examine Their Association with Long-Term Health Outcomes Following IR-Exposure?
Dicentrics	24	Yes
Acentric fragments	14	Yes
Micronucleus	32	Yes
Rings	14	No
Nucleoplasmic bridges	7	No
Sister chromatid exchanges	7	≈Yes
Translocations	6	≈Yes
Comet tail length	6	≈Yes
Comet tail moment	4	≈Yes
Comet score (DNA damage extent)	4	≈Yes
Premature centromere divisions	2	? (Yes)
Glycophorin A (GPA) mutant	1	? (Yes)
Leukocyte telomere length	1	? (Yes)
Copy number variation in AZFc region	1	? (Yes)
%DNA in the tail	3	? (Yes)
γ-H2AX foci	1	? (Yes)

Yes: direct evidence that this biomarker can be used as such; No: direct evidence that this biomarker cannot be used; ≈Yes: potential use; ?(Yes): candidate for further use.

**Table 4:** Detection limit dose and specificity for biomarkers.

Endpoints	Sensitivity	Specificity to IR
Dicentrics	50–100 mGy	High
Translocations	200–300 mGy	Good
Micronuclei	100–200 mGy	Good
Comet assay	50–100 mGy	Low
γ-H2AX foci	10 mGy	Good
Leukocyte telomere length	unknown	Low

## Data Availability

Not applicable.

## Supplementary Materials

The following are available online at https://www.mdpi.com/article/10.3390/ijms22147504/s1.
