# Supplementary material for: Biomarkers of Genotoxicity in Medical Workers Exposed to Low-Dose Ionizing Radiation: Systematic Review and Meta-Analyses
Source: Int J Mol Sci. 2021 Jul 13;22(14):7504. doi: 10.3390/ijms22147504 (PMC8304237; doi:10.3390/ijms22147504)
Supplement: Supplementary file 1 [file ijms-22-07504-s001.zip › Supplementary material.pdf]

Supplementary material

Figure S1: Forest plot of mean differences in NPB between IR exposed and unexposed workers, removing outlier

Study	Exposed N	Exposed Mean	Exposed SD	Unexposed N	Unexposed Mean	Unexposed SD	Hedges's g with 95% CI	Weight (%)
Gerić et al. (2019)	24	1.61	1.08	24	1.38	1.65	0.16 [-0.40, 0.72]	18.15
Gharibdousty et al. (2017)	35	1.02	0.02	35	0.85	0.37	0.64 [0.17, 1.12]	19.48
Maluf et al. (2001)	22	2.98	1.57	22	1.96	1.03	0.75 [0.15, 1.35]	17.44
Milic et al. (2015)	77	0.90	1.50	70	1.70	4.00	-0.27 [-0.59, 0.06]	21.75
Pajic et al. (2017)	201	0.75	0.85	201	0.23	0.47	0.76 [0.55, 0.96]	23.18
**Overall**							0.40 [-0.07, 0.88]	

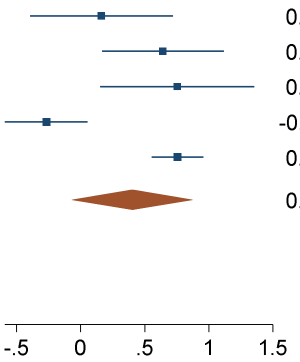

Heterogeneity: $\tau^2 = 0.24$, $I^2 = 86.76\%$, $H^2 = 7.55$

Test of $\theta_i = \theta_j$: $Q(4) = 30.21$, $p = 0.00$

Test of $\theta = 0$: $z = 1.66$, $p = 0.10$

Random-effects DerSimonian-Laird model
